# Revealing the global longline fleet with satellite radar

**DOI:** 10.1038/s41598-022-23688-7

**Published:** 2022-12-05

**Authors:** David A. Kroodsma, Timothy Hochberg, Pete B. Davis, Fernando S. Paolo, Rocío Joo, Brian A. Wong

**Affiliations:** 1 Global Fishing Watch, Washington, DC 20036 USA; 2 Marine Geospatial Ecology Lab, Nicholas School of the Environment, Duke University, Durham, NC USA; 3 SkyTruth, Shepherdstown, WV 25443 USA

**Keywords:** Marine biology, Conservation biology

## Abstract

Because many vessels use the Automatic Identification System (AIS) to broadcast GPS positions, recent advances in satellite technology have enabled us to map global fishing activity. Understanding of human activity at sea, however, is limited because an unknown number of vessels do not broadcast AIS. Those vessels can be detected by satellite-based Synthetic Aperture Radar (SAR) imagery, but this technology has not yet been deployed at scale to estimate the size of fleets in the open ocean. Here we combine SAR and AIS for large-scale open ocean monitoring, developing methods to match vessels with AIS to vessels detected with SAR and estimate the number of non-broadcasting vessels. We reveal that, between September 2019 and January 2020, non-broadcasting vessels accounted for about 35% of the longline activity north of Madagascar and 10% of activity near French Polynesia and Kiribati’s Line Islands. We further demonstrate that this method could monitor half of the global longline activity with about 70 SAR images per week, allowing us to track human activity across the oceans.

## Introduction

In recent years, advances in satellite technology have allowed large-scale tracking of industrial fishing vessels that use the Automatic Identification System (AIS). AIS devices broadcast the vessel’s GPS positions to help nearby vessels avoid collisions, and these AIS messages can be recorded by satellite constellations and used to estimate fishing activity^1^. Use of AIS devices, however, varies by region and fleet and is more common on larger vessels and those from wealthier nations^2^. This incomplete use of AIS might greatly limit our ability to estimate the total amount of fishing activity in a given region without accounting for the non-broadcasting, sometimes referred to as “dark,” vessels^3^. Moreover, non-broadcasting vessels appear to be more likely to engage in illicit activities. Of the few hundred vessels on the illegal, unreported, and unregulated vessel list^4^, only a handful broadcasted AIS in 2020 and 2021^5^, and a review of about 200 vessels with reported cases of forced labor showed that only about a quarter broadcasted AIS^6^. In recent years, the largest cases of illegal fishing were by fleets that mostly did not use AIS^7^.

Non-broadcasting vessels can be located, however, regardless of cloud cover and sunlight, by a well established spaceborne imaging technology, satellite Synthetic Aperture Radar (SAR) ^8,9^. Although the SAR technology for vessel detection is relatively mature, few studies have used it to estimate the size of fleets that cannot be identified through vessel GPS^7^. Several factors have prevented its widespread use, such as the high cost of SAR products, the complex data processing, and the fact that a single medium-resolution SAR scene covers less than 0.1% of the ocean. In addition, there is a major challenge in linking the SAR detections to specific broadcasting vessels, making it difficult to separate SAR detections corresponding to broadcasting and non-broadcasting vessels. Although AIS devices broadcast GPS positions every two seconds to three minutes, due to variable reception and intermittent satellite coverage, the time between the SAR image and the most recent GPS position for a given vessel may be several minutes to hours apart, also making it difficult to match AIS messages to specific SAR detections. Moreover, a typical SAR swath for vessel detection with a pixel size of 10–50 m will likely not see all vessels. The smaller a vessel is the less likely it is to be detected; consequently, if the average vessel size of a fleet is relatively small, the actual number of vessels in the region can be significantly larger than the number of vessels detected by SAR. While it is known that smaller vessels are less likely to be detected for a given SAR resolution^10^, this relationship has not been accounted for in estimating the number of vessels in non-broadcasting fleets.

We developed a method to estimate the size of non-broadcasting fleets by combining AIS data and SAR imagery, and we demonstrate its potential for monitoring the global pelagic longline fleet. We focus on the longline fleet because it is the most spatially widespread form of fishing, operating in a third of the global ocean^1^, and it is a fleet that is in urgent need of better monitoring. The fleet, which catches billions of dollars of tuna per year^11^, is lightly managed^12^, and only one out of every 20 vessels has an observer on board to document activity^13^, thus making it challenging to verify catch and bycatch. Some of the stocks targeted by these longline fleets are overfished, including yellowfin tuna in the Indian Ocean, albacore tuna in the eastern Pacific^14^, and southern bluefin tuna in the Southern Ocean^15^, and it will take continued coordinated efforts to rebuild these stocks. These fleets also have substantial bycatch risk, and they kill about 160,000 seabirds per year^16^. Longlines are also the major reason that albatross populations are declining, with 15 out of 22 species^17^ threatened with extinction^18^. Fish and seabirds are not the only ones at risk; more than half of drifting longline vessels were predicted to be at risk of forced labor in 2012–2018^6^, suggesting that thousands of fishers could also benefit from better oversight of this fleet.

**Figure 1:**
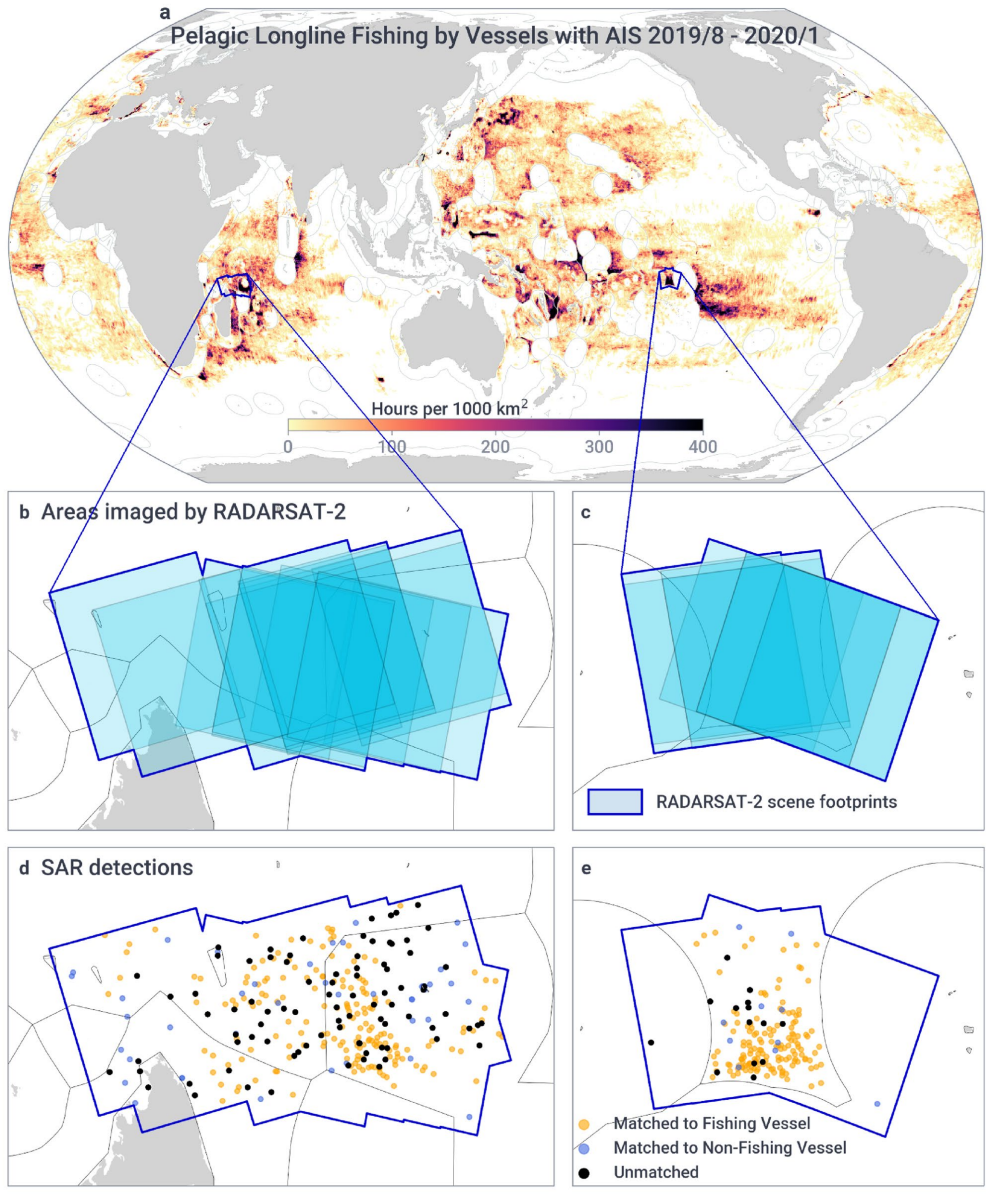
SAR detections were collected in two regions of intense longline fishing. (**a**) Global pelagic longline activity during our study period (Pacific: Aug–Sep/2019, Indian: Oct/2019 to Jan/2020), measured in hours of fishing per 1000 km^2^. (**b**) 15 images were acquired over the Indian Ocean north of Madagascar, including parts of the exclusive economic zones of Mauritius, Seychelles, and Madagascar. (**c**) Nine images in the Pacific focused on the high seas between Kiribati and French Polynesia, including parts of these countries' exclusive economic zones. (**d**, **e**) Detections of vessels from  SAR imagery, matched and unmatched to vessels broadcasting AIS. Maps generated using Python 3.9.6 and PySeas library (https://github.com/GlobalFishingWatch/pyseas).

We obtained processed AIS data from Global Fishing Watch’s database of vessels^1^ and obtained SAR imagery from the Canadian Space Agency RADARSAT-2 mission with respective vessel detections extracted by the satellite company KSAT (see “Methods”). The SAR imagery and detections covered two regions of the ocean with intense longline activity: the high seas between French Polynesia and the Line Islands Group of Kiribati in the Pacific Ocean, and the waters to the north of Madagascar in the Indian Ocean. In the Pacific, between August and September 2019, we acquired nine images covering a total area of about 429,000 km^2^, and in the Indian Ocean, between October 2019 and January 2020, we acquired 15 images covering about 754,000 km^2^ (Fig. 1). For each region, we matched vessels detected with SAR to vessels broadcasting AIS, assessed unmatched detections, and developed a probabilistic approach to estimate the number of non-broadcasting fishing vessels to account for potential vessels that were not detected by SAR. Using this approach, we then estimated how well we can monitor the global pelagic longline fleet with the proposed method.

## Results

### Quantifying non-broadcasting vessels with combined SAR and AIS

**Figure 2:**
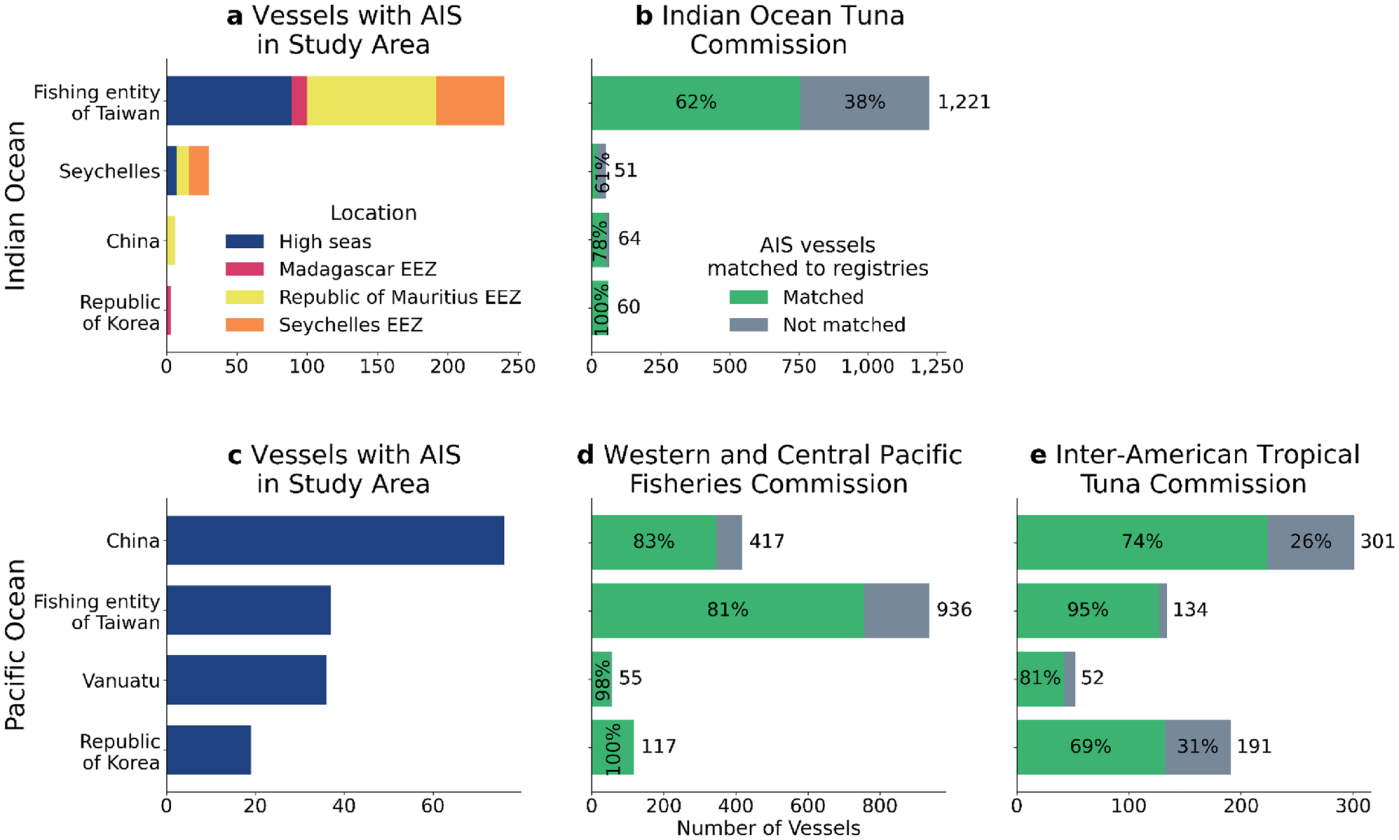
Regional fisheries management organization (RFMO) registries, matched to AIS data, suggest that 0–61% of the key fleets vessels are not broadcasting AIS in each ocean basin. Flag states of fishing vessels with AIS in each region (**a** and **c**) and vessels registered to RFMOs (**b**, **d**, **e**). In the Indian Ocean region (**a**), vessels with AIS were present in five different exclusive economic zones (EEZs), while in the Pacific (**c**) All vessels were in the high seas. Of the fleets that were present in the Indian Ocean, when compared to the Indian Ocean Tuna Commission (IOTC) registry (**b**), fishing entity of Taiwan and Seychelles had a significant number of vessels on the registry that were not identified in AIS (gray bar), suggesting that these vessels may be operating without AIS, while the other fleets did not. The Pacific Ocean region spans two RFMOs (**d**, **e**), the Western and Central Pacific Fisheries Commission and the Inter-American Tropical Tuna Commission, and each fleet present has registered vessels that don’t appear in AIS (gray bars). The fraction of non-broadcasting fleets, according to registries, varies from 0% for Korean vessels in the Indian Ocean to 39% for vessels in the Indian Ocean from the Seychelles.

The AIS data reveal the publicly available information of vessel activity in the region. According to AIS, around the time the 24 images were taken, a total of 203 unique vessels were present within the surveyed area. Of those vessels, 111 occurred in multiple images, giving a total of 529 instances of vessels that were likely within the footprints of the 24 satellite images. Around 94% in the Pacific region and 83% of the vessels in the Indian region were fishing vessels, all of which were drifting longlines. In the Pacific, all fishing vessel activity was in the high seas, with nearly all fishing activity represented by four flag states (Fig. 2c): China (45%), the fishing entity of Taiwan (22%), Vanuatu (21%), and the Republic of Korea (11%). In the Indian Ocean region, fishing activity (Fig. 2a) was represented mostly by the fishing entity of Taiwan (82%) and the Seychelles (10%), with few vessels from China (2%) and the Republic of Korea (1%), and a small percentage with unknown flag (6%). Most of the activity in this region was within the Mauritian exclusive economic zone, followed by the Seychelles, and then Madagascar. The AIS data did not show fishing activity in the Reunion exclusive economic zone.

To match these vessels broadcasting AIS to detections of vessels by SAR, we determined the likelihood a vessel would be at a given location (the location of a SAR detection) from the vessel’s GPS position closest to the time of the image, which could have been minutes to hours before or after the SAR image. We characterized patterns of vessel motion based on historical AIS data, and we produced probability maps to estimate the likelihood of a vessel location after (or before) a time interval given its speed, type, and trajectory (Figs. S1, S2). We found that this method far outperformed conventional approaches^19,20^ such as simple interpolation based on the vessel’s speed and course (Fig. S3, S4), while also providing a criterion for when to accept or reject matches between vessels’ GPS and SAR detections (Eq. 5).

**Figure 3:**
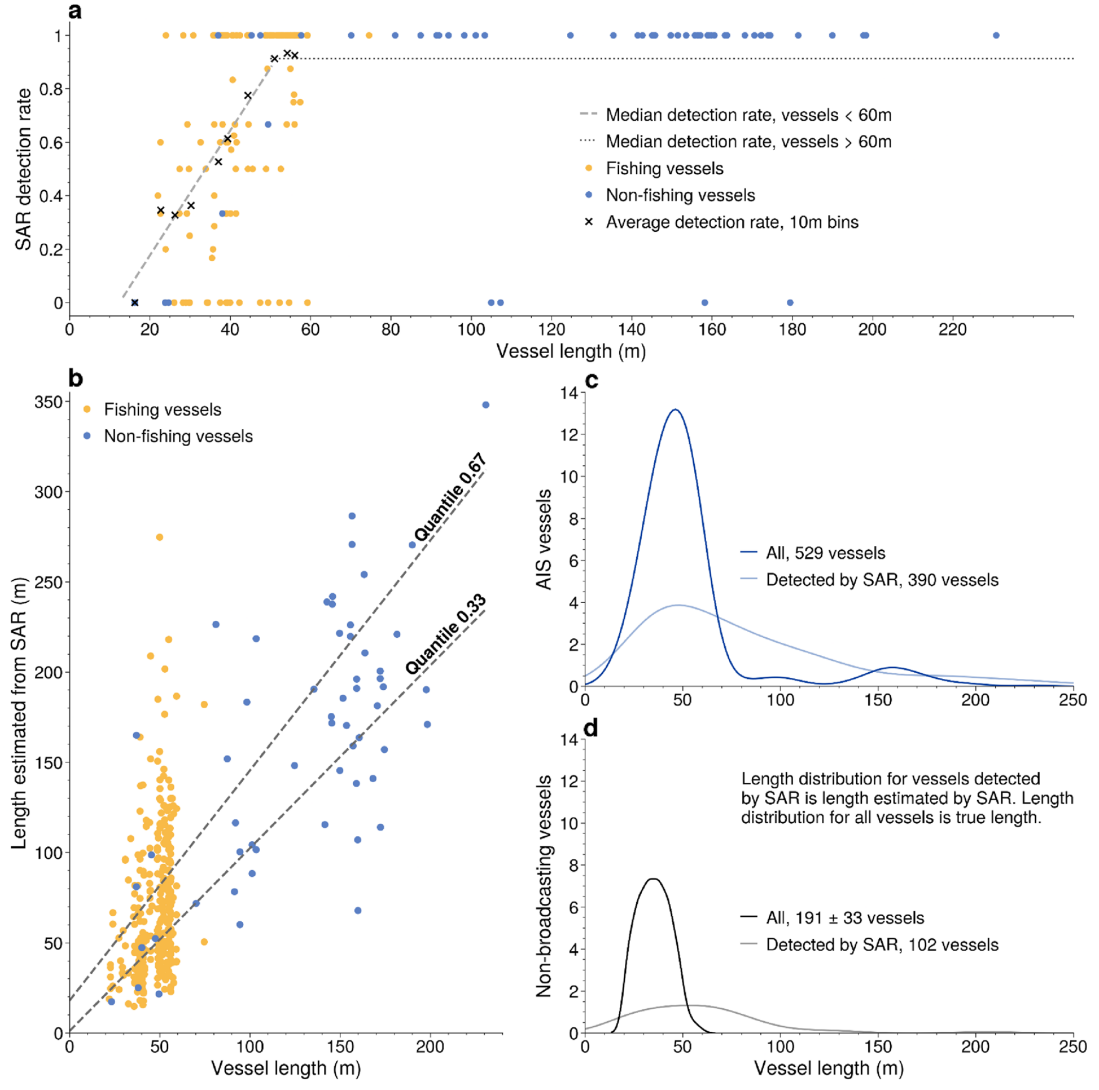
Relationships between SAR estimated length and actual length, and between length and SAR detection rate allow us to estimate the true distribution of non-broadcasting vessels. (**a**) For vessels under 60 m in length, the rate of detection with SAR increases linearly with length, while above 60 m it appears roughly constant, with only a few very large vessels undetected. Vessels that appeared in multiple scenes have fractional detection rates (between 0 and 1). (**b**) For vessels with AIS that matched to SAR detections, the length estimated from SAR shows high variability compared to the actual length of vessels, with quantile fits shown for the 33rd and 67th percentiles. (**c**) The distribution of actual lengths of the 529 vessels with AIS likely within the SAR scenes (dark blue curve) and the corresponding distribution of SAR estimated length for the 390 of these vessels detected by SAR (light blue curve). (**d**) The distribution of SAR estimated lengths of the 102 SAR detections of non-broadcasting vessels (gray curve), and, based on the relationships in A and B, the estimated most likely distribution of actual non-broadcasting vessels (black curve).

**Figure 4:**
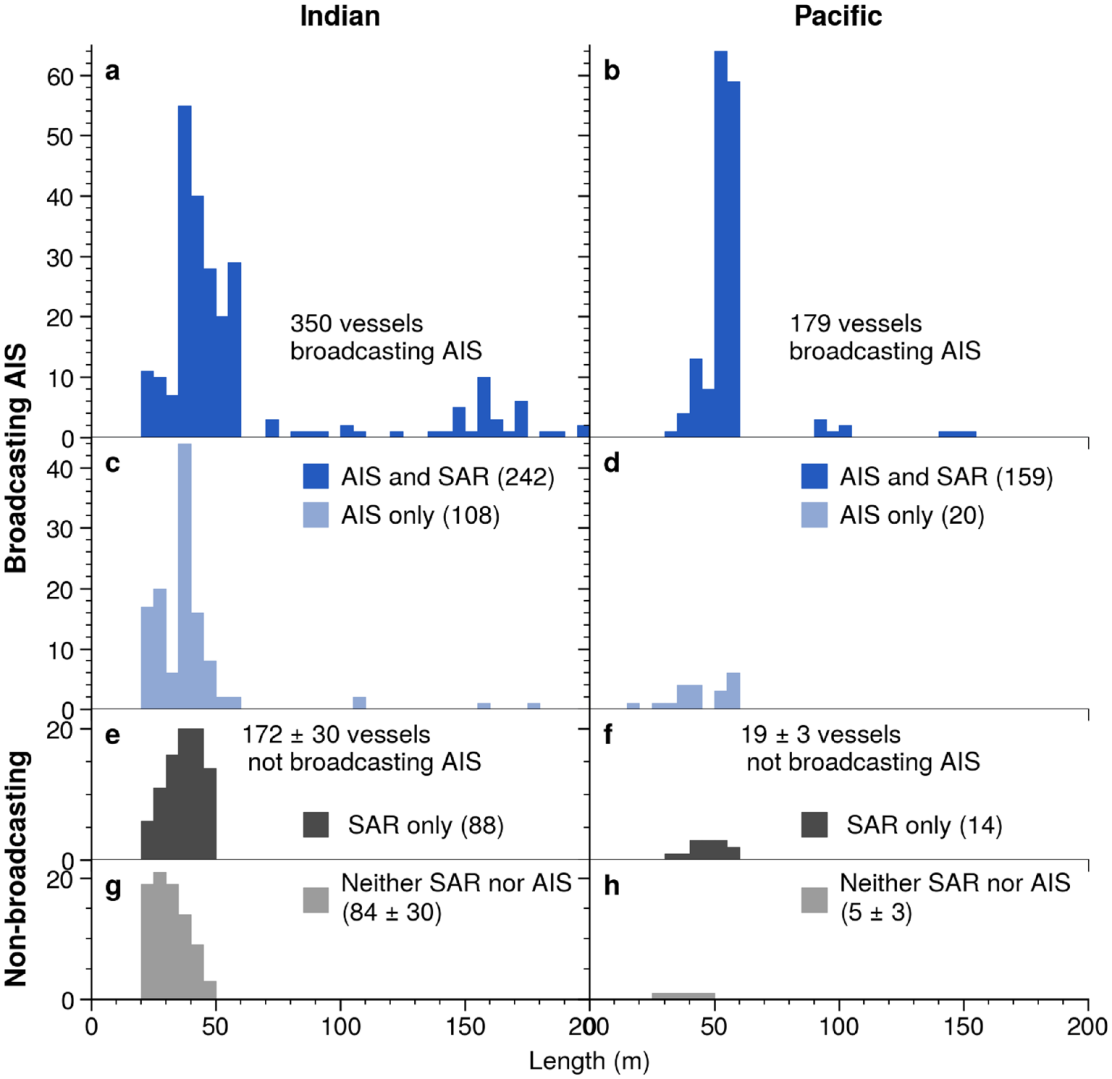
Vessels are detected by AIS, SAR, both, and neither. (**a**, **b**) SAR detections that matched to AIS in the Indian and Pacific regions. (**c**, **d**) Vessels broadcasting AIS in each region that were likely within the image footprints but not detected by SAR. (**e**, **f**) Unmatched SAR detections, representing non-broadcasting vessels. (**g**, **h**) The most likely distribution of vessels detected by neither SAR nor AIS.

By using this matching system, we could identify SAR detections that were non-broadcasting vessels and AIS vessels that were not detected by SAR. In the 24 SAR images, 493 vessels were detected, with 320 vessels in the Indian Ocean and 173 in the Pacific Ocean. Of these, 391 (374–394) matched to AIS vessels (Fig. 4a,b), while 102 (99–109) did not match to AIS vessels (Fig. 4e,f), with the majority 88 (86–93) located in the Indian region. The range of unmatched SAR reflects the fact that a few SAR detections could not be unambiguously matched to AIS (Fig. S3). In addition to many SAR detections not matching to AIS, many vessels broadcasting AIS were not detected by SAR (Fig. 4c,d). About a quarter (25–27%) of the vessels broadcasting AIS, almost all of which were small vessels, were not detected (97% of undetected vessels with AIS were < 60 m in length). For those smaller vessels, the detection rate appeared to decrease linearly, with vessels 60-m long detected over 90% of the time and vessels 20-m long about 20% of the time (Fig. 3a).

### Estimating the non-broadcasting fleet by accounting for undetected vessels

The number of non-broadcasting vessels in a region consists of the number of unmatched (relatively large) vessels plus an unknown number of non-detected (relatively small) vessels. Since the SAR detection algorithm estimates the lengths of these unmatched (non-broadcasting) vessels, we can use the relationship between the likelihood of detection and vessel length (Fig. 3a) to estimate the most likely number of actual non-broadcasting vessels in the region (see “Methods”). A major challenge, however, is the fact that the length estimates from RADARSAT-2 are noisy and have a large spread (Fig. 3b). To address this challenge, we modeled the relationship between estimated lengths (from SAR, henceforth SAR lengths) and actual lengths (from AIS). We then adjusted our estimated lengths using this model (henceforth modeled lengths) so as to minimize the difference between the SAR lengths and the modeled lengths (Eqs. 6–9).

By combining the AIS data with the SAR detections and developing these probabilistic models, we estimate 172 ± 30 (90% CI) non-broadcasting vessels in the Indian Ocean. The vessels were likely almost all smaller than 60 m. Non-broadcasting vessels in the Pacific were slightly larger than the Indian, with the median non-broadcasting vessels in the Indian between 30 and 35 m, and between 40 and 45 m in the Pacific (Fig. 4e–h, Figs. S5,6). Assuming that the proportion of fishing to non-fishing vessels is the same as in the AIS data (for vessels under 60 m), between 32 and 40% of the fishing vessels in the Indian Ocean region were not broadcasting AIS. In the Pacific, the 14 unmatched SAR detections suggest 19 ± 3 non-broadcasting vessels, constituting 10% to 12% of the fishing activity (Fig. 4h).

### Monitoring the longline fishing fleet globally with SAR

**Figure 5:**
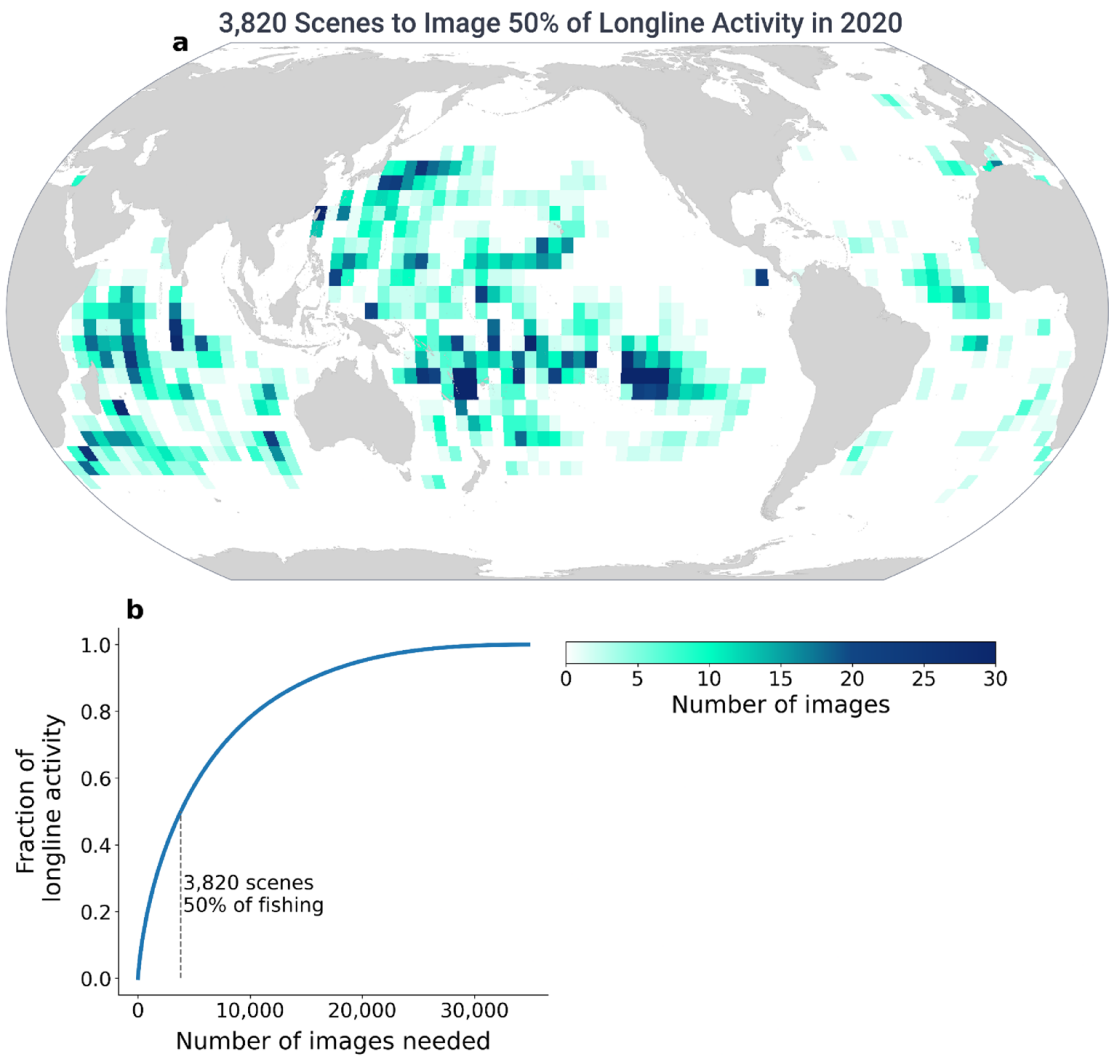
Half of the longline activity can be imaged by sampling only a small fraction of the ocean. Spatially (**a**) and temporarily (Fig. S7), longline activity is highly concentrated. Dividing the world into a four-by-four degree grid by 52 weeks for the calendar year 2020, half of the longline activity is in just 10% of the total cells where longline activity occurs (**b**). To image 50% of the longline activity, one would need 3820 scenes, distributed appropriately in space (**a**) and time (Fig. S7). Map generated using Python 3.9.6 and PySeas library (https://github.com/GlobalFishingWatch/pyseas).

To estimate how much of the longline fleet could be monitored with this type of imagery, we divided the world into four by four degree cells, which is roughly the size of one Radarsat-2 image, and divided the year into 52 weeks. Then, we analyzed longline fishing activity for 2020 based on AIS data, and identified the minimum number of these cells that would be needed to capture a given percentage of the fishing activity. Assuming that fishing by non-broadcasting vessels follows a similar pattern to fishing with AIS, it is possible to image about half of the longline activity with as few as 70 RADARSAT-2 images per week, or about 3800 per year, (Fig. 5, Fig. S7).

## Discussion

This study demonstrates the value of combining AIS and medium-resolution SAR. By combining the two sources, we can estimate the total number of vessels operating, including many vessels that were detected by neither AIS or SAR. Although higher resolution SAR imagery would have likely detected more vessels, the images would have covered less area, limiting our monitoring. The area we imaged, almost 1.2 million square kilometers, is roughly four times the area of the next largest study for monitoring fleet size with SAR^7^, and this is the first study of its kind to address drifting longlines. This type of monitoring will be critical for the open ocean. Over the past decades, extractive industries like fishing, mining, and shipping have expanded beyond national jurisdictions into the high seas, while tools to monitor and regulate these activities have lagged behind^21^.

We can now estimate the actual footprint of longline activity. In both regions, we found that non-broadcasting and broadcasting vessels showed similar spatial distributions. In the Pacific, almost all vessel activity (from both AIS and SAR) is in the high seas, with almost no activity in French Polynesian waters or Kiribati, suggesting there is no large hidden “dark fleet” operating in French Polynesia or Kiribati. Similarly, in the Indian Ocean, we see no GPS positions or SAR detections in the northern Reunion EEZ, also suggesting that there is no large untracked fleet operating there. In both regions, while AIS may not cover all vessel activity, it provides, roughly, the correct spatial footprint of fishing.

Our method also lets us identify the lengths of the non-broadcasting fleets, which is useful because vessel size corresponds to fishing effort (larger vessels usually catch more fish) and because it can identify vessels out of compliance with AIS regulations. We found that the lengths of non-broadcasting vessels were larger than expected. Many vessels over 40 m were not broadcasting AIS, with about a quarter of the non-broadcasting vessels in the Indian Ocean being over this length and two thirds of the non-broadcasting vessels in the Pacific. A previous analysis^1^ suggested that globally almost all fishing vessels larger than this length have AIS devices. Either these large vessels are operating with their AIS disabled, or there are more large vessels without AIS than previously known.

It is possible that some of these larger non-broadcasting vessels are out of compliance, although most fleets do not have clear requirements. For instance, Korean vessels larger than 10 tons (10 ton vessels are almost always smaller than 20 m) are required to have AIS^22^. There are no known mandates for Seychelles or Chinese vessels to have AIS, and at the time of this study, the fishing entity of Taiwan had no mandate for AIS, although as of January 2022 it is required on all vessels over 20 tons (although there appears to be no penalty for not having AIS on^23^). It is worth noting that there is no specific international mandate for AIS because the international regulations requiring AIS, set by the International Maritime Organization (IMO), specifically exempts fishing vessels^24^—and this situation could be remedied by stricter international regulations.

A key question relates to the identity of these non-broadcasting vessels. To uncover their identity, we reviewed the list of vessels authorized to fish in the Indian Ocean Tuna Commission (IOTC), the Western and Central Pacific Fisheries Commission (WCPFC), and the Inter-American Tropical Tuna Commission (IATTC). Any vessel fishing in these regions should be registered with one of these lists^25^, but being on the list does not guarantee the vessel was active in the region during the time of our survey. Of the fleets that were present in the Indian Ocean region, only the Seychelles and fishing entity of Taiwan (39% and 38% of all fishing vessels, respectively) had a sizable number of vessels that were not broadcasting AIS according to the IOTC registry (Fig. 2b). Given that vessels from the fishing entity of Taiwan and Seychelles account for 90% of the fishing activity in the study area, it is plausible that all detected non-broadcasting vessels belong to those fleets.

The fishing entity of Taiwan, associated with a large number of non-broadcasting vessels in the Indian Ocean, is of particular concern. Vessels with this flag have been identified as a major culprit of human rights violations at sea including forced labor and human trafficking of migrant workers^26^. In 2019, for example, nearly 30,000 migrants from the Philippines and Indonesia worked onboard vessels flagged to the fishing entity of Taiwan^27^. Given the high risk of these activities, it may behoove their flag state to require all vessels to share their locations publicly and demonstrate their activities clearly.

The Pacific Ocean region has a different management challenge, as our study region is in an overlapping area between the IATTC and the WCPFC. This overlap leads to some uncertainty regarding vessel registries, as vessels fishing for tuna need to be registered to only one of the regional fisheries management organizations (RFMOs) to fish in this part of the ocean^28^. Of the five fleets that were present, Republic of Korea, Vanuatu, French Polynesia, the fishing entity of Taiwan, and China, all had at least some vessels that were not registered (Fig. 2d,e).

The overlapping jurisdictions make it difficult to gauge compliance and highlights the need for independent sources of information such as SAR. In theory, vessels may be in compliance with regulations because they share their GPS locations with the commissions, using private GPS systems via Vessel Monitoring Systems (VMS). In practice, however, the regulations are somewhat unclear whether vessels in this region have to report to both tuna commissions, and some regulations state that fishing vessels have to register and follow the regulations of only one of them^28^. This ambiguity is reflected in catch data: for 2019, the WCPFC and IATTC provided different estimates of the amount of fishing for each fleet in this part of the ocean. For example, the WCPFC data suggests that in this region Korea placed 36% as many hooks as reported to the IATTC and caught 40% of the reported fish to the IATTC (Fig. S7). This data mismatch leads to ambiguity over authorization and fishing activity—ambiguity that could be mitigated by all the vessels present broadcasting their movements.

Although our study areas accounted for less than one percent of the global ocean, we demonstrated how we could monitor the majority of longline activity in the ocean with a modest amount of imagery. The roughly 70 images required per week to monitor half of the global longline fleet is only a small fraction of the total capacity of RADARSAT-2, the satellite used in this study. Deploying the methods outlined in this paper (with the code in our public GitHub repository) and imaging the areas of the ocean suggested in Fig. 5 and S7, could help estimate the true extent of longline fishing across the ocean; and potentially highlight areas where a significant fraction of the activity is unmonitored to better inform management agencies. Moreover, these methods can be scaled and applied to any region where SAR imagery is available, and can also be applied to all non-fishing, ocean-going vessels. As human activity continues to expand into waters far from shore, this information will be critical to managing the world’s largest shared resource, the ocean.

## Methods

To estimate the total number of non-broadcasting vessels, including those that were not detected by SAR, we: (1) obtained SAR detections of vessels from RADARSAT-2 and the corresponding vessel lengths as estimated from the SAR image; (2) processed a global feed of AIS data to identify every broadcasting vessel that should have appeared in the SAR images at the moment the images were taken; (3) developed a novel technique to determine which vessels in AIS matched to detections in SAR, which AIS vessels were not detected by SAR, and which SAR detections represented non-broadcasting vessels; (4) after matching SAR to AIS, we could then (a) model the relationship between a vessel’s actual length and the length as estimated by the SAR image (Fig. 3b) and (b) model the relationship between the likelihood that a vessel is detected and its length (Fig. 3a); and (5) finally, we combined these relationships to develop an estimate of the number and lengths of non-broadcasting vessels in the region.

### SAR imagery and vessel detections

Working with the satellite company Kongsberg Satellite Services (KSAT), we tasked the Canadian Space Agency’s satellite RADARSAT-2 to acquire SAR images from its ship detection mode (DVWF mode, GRD product), with a pixel size of about 40 m and a swath width over 400 km (19). These images were processed following standard procedures for GRD products (e.g. applying radiometric calibration and geometric corrections)^29,30^. Vessel locations were extracted from the images with the widely used ship detection algorithms, which discriminates objects at sea based on the backscatter difference (pixel values) between the sea clutter and the targets^31^. Vessel lengths were estimated by measuring distances directly on the images with the aid of a graphical user interface tool^31^.

### Identifying Vessels using AIS

In each region, AIS data, obtained from satellite providers ORBCOMM and Spire, were processed using Global Fishing Watch’s data pipeline^1^. The identities and lengths of all AIS devices that operated near the SAR scenes in both space and time were first obtained using Global Fishing Watch’s database^1^. To be sure vessels were identified correctly, two analysts reviewed the tracks of every AIS device in each region.

In both regions, it is common practice for fishers to put AIS beacons on their longlines, likely to aid in retrieving them, meaning that many AIS devices were longline gear and not vessels. Because gear outnumbered vessels by several-fold, it was critical to differentiate gear and fishing vessels. In the Indian Ocean, 521 unique AIS devices associated with gear were detected that were likely within the SAR scenes, and 390 unique AIS devices associated with gear in the Pacific that were likely within the SAR scenes. Transponders were determined to be associated with gear by inspecting the name broadcast in the AIS messages (gear frequently broadcasts one of several standard names and/or a voltage reading) and classification using the Global Fishing Watch vessel classification algorithm^1^. Most gear also had an MMSI number (unique identifier number for AIS) that started with 1, 8, or 9 or broadcast names that signified gear. We eliminated all gear from the analysis because (1) these gear buoys have reflectors that are only ~ 1 m in size, and they should not be visible in ~ 40 m resolution SAR images, and (2) we found that gear matched to SAR detections only when traveling faster than 2 knots (and thus was on the deck of a boat); of 159 instances of gear in scenes where the gear was traveling slower than two knots, zero matched to a radar detection (Fig. S9).

### Generating probability rasters for matching AIS to SAR

Most AIS positions did not correspond to the exact time when the SAR images were taken. Hence, to determine the likelihood that a vessel broadcasting AIS corresponded to a specific SAR detection, we first developed probability rasters of where a vessel was likely to be minutes before or after a GPS position was recorded (Figs. S1,S2). We mined one year of global AIS data, including roughly 10 billion GPS positions, and computed these rasters for six different vessel classes (trawlers, purse seines, tug, cargo or tanker, drifting longlines, and others) and considered six different speeds (1, 3, 5, 7, 9, and 12.5 knots) and 36 time intervals (− 448, − 320, − 224, − 160, − 112, − 80, − 56, − 40, − 28, − 20, − 14, − 10, − 7, − 5, − 3.5, − 2.5, − 1.5, − 0.5, 0.5, 1.5, 2.5, 3.5, 5, 7, 10, 14, 20, 28, 40, 56, 80, 112, 160, 224, 320, and 448 min).

For example, we queried a year of AIS data to find every example of where a tugboat had two positions that were 10 min apart from one another when the vessel had been traveling at 10 knots at the first position. We then recorded each of these locations relative to the location the vessel would have been if it traveled in a straight line, with x coordinates being in the direction of travel and the y coordinates being perpendicular to the direction of travel. When collected for hundreds of thousands of examples across the AIS dataset, the result is a heatmap of where tug boats are located 10 min after a position when it was traveling at 10 knots. The raster is centered on a point that is the extrapolated position of the vessel based on its speed. For instance, the purse seine raster that corresponds to a vessel traveling between 6 and 8 knots between 96 and 128 min after the most recent position is centered at a point that is 13.1 km (7 knots × 112 min) straight ahead of the direction the vessel was traveling. Figure S1 shows samples of these rasters for different vessels.

We built rasters of 1000 by 1000 pixels for each vessel class and time interval, with the area covered by the raster dependent on the time interval (longer time intervals imply longer traveled distances, covering more area). The scale of each pixel was given by:

1 $${\text{pixel}}\;{\text{width = max(1, }}\Delta {\text{m) / 1000}}$$

where *Δm* is the time interval in minutes, and pixel width is measured in km. Thus, if the *Δm* is under one minute, the entire raster is one kilometer wide with each pixel one meter by one meter. If the time is 10 min, then each pixel is 10 m wide, and the entire raster is 10 km by 10 km.

Since the pixel width varies between rasters, the units of the rasters are probability per km^2^, thus summing the area of each pixel times its value equals one. Six vessel classes with 36 time intervals for each and six speeds led to 1296 different rasters. This probability raster approach could be seen as a utilization distribution^32^—for each vessel class, speed and time interval—where the space is relative to the position of the individual.

### Combining probability rasters to produce a matching score

For a few vessels (~ 4%) there was only one AIS position available before or after the scene. This resulted from a long gap in the AIS data due to poor reception, a weak AIS device, or cases where the vessels disabled their AIS. For these vessels, we used the raster values for a single position. For the vast majority of vessels, however, there was a GPS position right before and after the scene, and thus two probability rasters. We used two methods to combine these probability rasters to obtain information about the most likely location:

#### Multiply and renormalize the rasters

To multiply the rasters, we interpolated the raster values, using bilinear interpolation, to a constant grid at the highest resolution between the before and after rasters. Then, we multiplied the values at each point and renormalized the resulting raster (Fig. S2):

2 $$p_{i} = \frac{{p_{ai} \cdot p_{bi} }}{{\mathop \sum \nolimits_{k = 0}^{N} p_{ak} \cdot p_{bk} \cdot da}}$$

where *p*_*i*_ is the probability in vessel density per km^2^ at location *i*, *p*_*ai*_ is the value of the raster before the image, *p*_*bi*_ is the value of the raster after the image. The denominator is the sum of all multiplied values across the raster, scaled by the area of each cell, *da*.

#### Weight and average the rasters

 For this method, we weighted the raster by the squared value of the probabilities of that scene. This has the effect of giving the concentrated raster a higher weight, thus weighting higher the raster that is closer in time to the image:

3 $${w}_{a}=\sum_{k=0}^{N} {p}_{ak}^{2}\cdot da$$

and the weighted average at location *i* is:

4 $${p}_{i}=\frac{{p}_{ai}\cdot {w}_{a}+{p}_{bi}\cdot {w}_{b}}{{w}_{a}+{w}_{b}}$$

where *w*_*a*_ is the weight for raster *a*, *w*_*b*_ the weight for raster *b* (calculation analogous to w_a_’s in Eq. 3), *p*_*i*_ is the probability in vessel density per km^2^ at location *i*.

To determine whether we should multiply (Eq. 2) or average (Eq. 4) the probabilities, we compared the performance of these two metrics against a direct inspection of the detections. We found that at short intervals, multiplying the rasters and renormalizing often made probability values extremely small (< 1 × 10^–4^) despite direct inspection by an expert analyst suggesting there was a high likelihood of match (i.e. that the probability of a vessel being in location *i* should be high). Overly small values of multiplied probabilities could happen, for example, when there is a position one minute before the image and another one minute after, in which case both rasters contain almost the same information and, therefore, are virtually identical. This is analogous to squaring the probability values of one of the rasters (Fig. S3). On the other hand, at longer time ranges the averaging scheme tended to overestimate the matching rates of some highly unlikely matches. We established an ad hoc rule based on our data: when the closest position was within 10 min of the image, we used a weighted average (Eq. 4), and multiplied (Eq. 3) at longer time ranges.

### Ranking and matching potential SAR to AIS pairs

We computed a matrix of scores of potential matches between SAR and AIS. We then greedily assigned matches, i.e. the AIS-SAR pair with the highest score (highest *p*_*i*_ from Eq. 4) is selected as a match and the corresponding row and column are removed from the matrix. Then the next highest score is assigned and so on until all pairs have been assigned. In cases where a single AIS vessel presented an equally high matching score to multiple SAR detections, or vice versa, we manually reviewed the matching pairs (mostly for cases where the scores were within a factor of 100 of one another).

### Choosing a threshold for accepting SAR to AIS matches

A major challenge is deciding when the probability score is too low to accept a match. We reviewed matches with two independent analysts, classifying the AIS to detection pairs as: “likely matches” when there was a high likelihood of matching, “potential matches” when it was not possible to determine, and “unlikely matches” when there was a low probability of matching. Comparing these classifications with different score thresholds, we found that a matching score between 5 × 10^–6^ and 1 × 10^–4^ vessel km^−2^ provided a reasonable threshold for accepting/rejecting a match. Only a few pairs had scores between this range; most potential matches had higher or lower scores, and thus our results are not very sensitive to the different thresholds. Only nine likely matches (2% of total) between SAR detections and vessels broadcasting AIS were ambiguous, meaning that a vessel broadcasting AIS could have matched to multiple SAR detections or, conversely, a SAR detection could have matched to multiple vessels broadcasting AIS.

This analysts’ judgment is roughly in line with a theoretical estimate. Given a single SAR detection and AIS vessel, there are three possible options: (1) the detection represents the AIS vessel, (2) the AIS vessel was not detected by SAR and the detection represents a non-broadcasting vessel, or (3) the AIS vessel was not detected by SAR and the detection represents a false positive. We should match the SAR detection to the AIS vessel if the probability of (1) is greater than the probability of (2) and (3):

5 $${\text{Match}} \, {\text{detection}} \, {\text{to}} \, {\text{AIS}} \, {\text{if}} \, {p}_{v}\cdot {p}_{d} > {d}_{d}\cdot {p}_{d} + {p}_{f}$$

where ${p}_{v}$ is the probability density of the vessel presence at the location of the SAR detection (the score listed above), ${p}_{d}$ is the probability that the vessel is detected by SAR, ${d}_{d}$ is the density of non-broadcasting vessels in the region, and ${p}_{f}$ is the density of false detections in the scene. The greater ${p}_{d}$, the more dark vessels there are in a scene, and the more likely it is that any given detection is a dark vessel instead of a vessel broadcasting AIS. The right-hand side of the equation ${d}_{d}\cdot {p}_{d} + {p}_{f}$ should roughly equal the number of detections per unit area that do not match to AIS in the region. In other words, the probability of the vessel with AIS being at that specific location and detected by SAR (left side of the equation) should be greater than the probability of a dark vessel or a false detection at that location (right side of the equation).

The total number of unmatched vessels in each studied region normalized by total area covered gives a density of non-broadcasting vessels of 2.6–2.8 × 10^–5^ vessels km^-2^ (Indian Ocean) and 6.8–7.2 × 10^–6^ vessels km^−2^ (Pacific Ocean), similar to the thresholds estimated by analysts. For the most likely number of matched vessels, we use a threshold that is halfway between the higher and lower bound of the analyst (5 × 10^–5^ to 1 × 10^–4^), 2.5 × 10^–5^ which is also roughly equal to the theoretical estimate of the Indian Ocean.

This threshold approach performed significantly better than a metric based on the distance between the SAR detection and the most likely location of the vessel, where the likely location is based on extrapolating speed and course of the position closest in time to the image (Fig. S4).

### Determining whether a vessel with AIS was within a scene

Vessel positions from AIS are usually available before and/or after the SAR images, and sometimes it is unclear if a vessel should have been within the scene footprint at the time of the image.

To estimate the probability that a vessel (with AIS) was within a scene, we used the multiplied probability raster, summing the values inside the scene boundaries. This provides an estimate of the likelihood that the vessel was within the scene footprint at the time of the image. We applied this to every vessel that had at least one AIS position within 12 h and 200 nautical miles of the scene footprint. The vast majority of vessels were either *very likely* inside or outside the scene footprints, with 516 vessels having a probability of > 95% and only 16 having a probability between 5 and 95%. We filtered out all vessels that were definitely outside of the image footprint before matching.

### Estimating the likelihood of detecting a vessel with SAR

The AIS data show that not all vessels broadcasting AIS were captured by the RADARSAT-2 images (Fig. 3a). Using the known lengths of detected vessels with AIS, we estimated the likelihood of detecting a vessel with SAR as a function of vessel length (Fig. 3a). For vessels shorter than 60 m, we approximated the detection rate as a linear function. Treating each vessel as an individual detection, we fitted the 50th percentile using quantile regression to approximate the detection rate. For vessels above 60 m, we assumed a constant detection rate as very few vessels above this length did now show up in the SAR images. Of the 46 unique vessels larger than 62 m, 42 were detected, implying a detection rate of ~ 91%. Given that it is highly likely that large vessels will be captured by medium-resolution SAR imagery, we manually reviewed these cases to confirm that they were (almost surely) inside the scene footprints at the time the images were taken.

We should note that the probability of detecting a vessel in SAR also depends on the sea state, incidence angle, polarization, material of the vessel, and orientation of the vessel. We are unable, however, to measure these effects directly so we cannot explicitly model these effects.With sufficient scenes, these effects should be randomly distributed across our scenes, so they likely account for some of the variability in detectability and the inaccuracy in our length estimates from SAR.

### Estimating the number and length of non-broadcasting vessels

Because SAR does not detect all vessels, and because the length as estimated by SAR can be incorrect, there are many possible distributions of actual non-broadcasting vessels that could have produced the distribution of unmatched SAR detections that we found in the scenes. To estimate the most likely such distribution, we built a model to combine the two key relationships—between vessel length and likelihood of detection, and between vessel length and the length as estimated by SAR. This model allowed us to estimate, based on the number and distribution of SAR vessels, the likely number and distribution of actual vessels present (Fig. 3c,d).

We binned the likelihood of vessel detection as a function of length into 1 m intervals, yielding a vector $\alpha$ of length 400. We also binned into 1 m intervals the population of lengths of all detected vessels ($\ell_{D}$) as reported by AIS (i.e. number of vessels at each length bin), the population of expected SAR lengths ($\ell_{E}$), and the population of lengths of all vessels ($\ell_{A}$, the quantity we wish to estimate). Thus, $\ell_{D}$ can be expressed as the product of $\alpha$ and $\ell_{A}$:

6 $$\ell_{D} = {\upalpha } \odot \ell_{{\text{A}}}$$

where $\odot$ is the element-wise product. We then estimated a matrix $L_{{}}$ that relates $\ell_{D}$ to $\ell_{E}$.

7 $$\ell_{E} = L\ell_{D}$$

where each element $L_{ij}$ represents the probability that a vessel with length in bin *j* would be estimated by SAR to be of length in bin *i*. We calculated these probabilities as lognormal probability density functions, with one distribution per column. To estimate the scale and shape parameters of these distributions, we first fitted a quantile regression using the (non-binned) lengths from AIS of detected vessels as the predictor for the lengths reported by SAR. Assuming that the predicted ^1/3^ and ^2/3^ quantiles (as shown in Fig. 3a) represent the quantiles of a lognormal distribution, allow us to calculate the shape and scale parameters. We chose a lognormal distribution because: 1) the variable of interest, length, was always greater than zero, 2) the population of lengths was skewed towards larger values, and 3) there is an explicit and relatively simple relationship between the lognormal quantiles and the shape and scale parameters that simplified the calculations.

Combining Eqs. (6) and (7) provides a relation between $\ell_{A}$ and $\ell_{E}$:

8 $$\ell_{E} = {\text{L}}\left( {\alpha \odot \ell_{A} } \right)$$

To estimate ${\mathcal{l}}_{A}$ we minimized an objective function $O({\mathcal{l}}_{E},{\mathcal{l}}_{o})$ between the vector of expected counts binned by length (${\mathcal{l}}_{E}$) and the vector of counts observed in SAR binned by length (${\mathcal{l}}_{o}$). For this objective function, we chose the sum of the Kolmogorov –Smirnov distance between length distributions and the squared difference of the total numbers of detections. The first term controls the shape of the resulting distribution while the second one controls the magnitude. Specifically:

9 $$O\left( {\ell_{E} ,\ell_{o} } \right) = \max \left( {\left| {C_{E} - C_{O} } \right|} \right) + \left( {T_{E} - T_{O} } \right)^{2}$$

where:

$$T_{x} = \mathop \sum \limits_{ } \ell_{x}$$

$$D_{x} = \ell_{x} /T_{x}$$

$$C_{x} = cumsum\left( {D_{x} } \right)$$

### Assessing the uncertainty in the estimation

To test how accurately our approach predicts the correct number of vessels, we performed a bootstrap simulation. We computed the vector $\alpha$ and the matrix L from a random subset of vessels with AIS that had a high confidence (> 95%) of appearing within the scenes. We then used our method on the SAR detections that matched the remaining vessels to predict the number of vessels they corresponded to ($\ell_{\text{A}}$). By running 10,000 experiments we found a mean absolute percent error of + − 9% (Figs. S5 and S6). This provides a rough estimate of the uncertainty in our prediction due to the estimation process itself. We used the distribution of these samples to estimate the 90% confidence interval that we report with our estimates. We note that this uncertainty refers to the parametrization of the model and there may be other sources of error, such as the possibility that vessels without AIS have different radar properties (e.g. made out of materials with different reflectiveness), that we did not account for in our model.

### Catch and effort data in the overlapping area between WCPFC and IATTC

We downloaded gridded effort and catch data from the WCPFC and IATTC websites, and compared the reported number of hooks and catch from September to December of 2019 for the area between − 140 to − 150 longitude and − 5 to − 15 latitude, a bounding box that contains our study region in the Pacific and which is entirely within both the WCPFC and IATTC convention zones. We found that the reported number of hooks for Korea is three times higher for the IATTC as it is for the WCPFC (Fig. S7), and the numbers of hooks also disagree by more than 10% for most other flag states. Catch is also 2.5 times higher for IATTC than for WCPFC for Korea as well, with catch also differing by more than 10% for most other flag states. This finding suggests that the different RFMOs may not be accounting for the same vessels in the overlap region between the two RFMOs.

## Supplementary Information

Supplementary Information.

## Data Availability

The code and data to reproduce these analyses is available at https://github.com/GlobalFishingWatch/paper-longline-ais-sar-matching.
