# Supplementary material for: Revealing the global longline fleet with satellite radar
Source: Sci Rep. 2022 Dec 5;12:21004. doi: 10.1038/s41598-022-23688-7 (PMC9722684; doi:10.1038/s41598-022-23688-7)
Supplement: Supplementary file 1 — Supplementary Information. [file 41598_2022_23688_MOESM1_ESM.pdf]

Supplementary Materials for

Revealing the Global Longline Fleet with Satellite Radar

David A. Kroodsma, Timothy Hochberg, Pete B. Davis, Fernando S. Paolo, Rocío Joo, Brian A. Wong

*Corresponding author. Email: david@globalfishingwatch.org

This PDF file includes:

Supplementary Figures S1 to S9

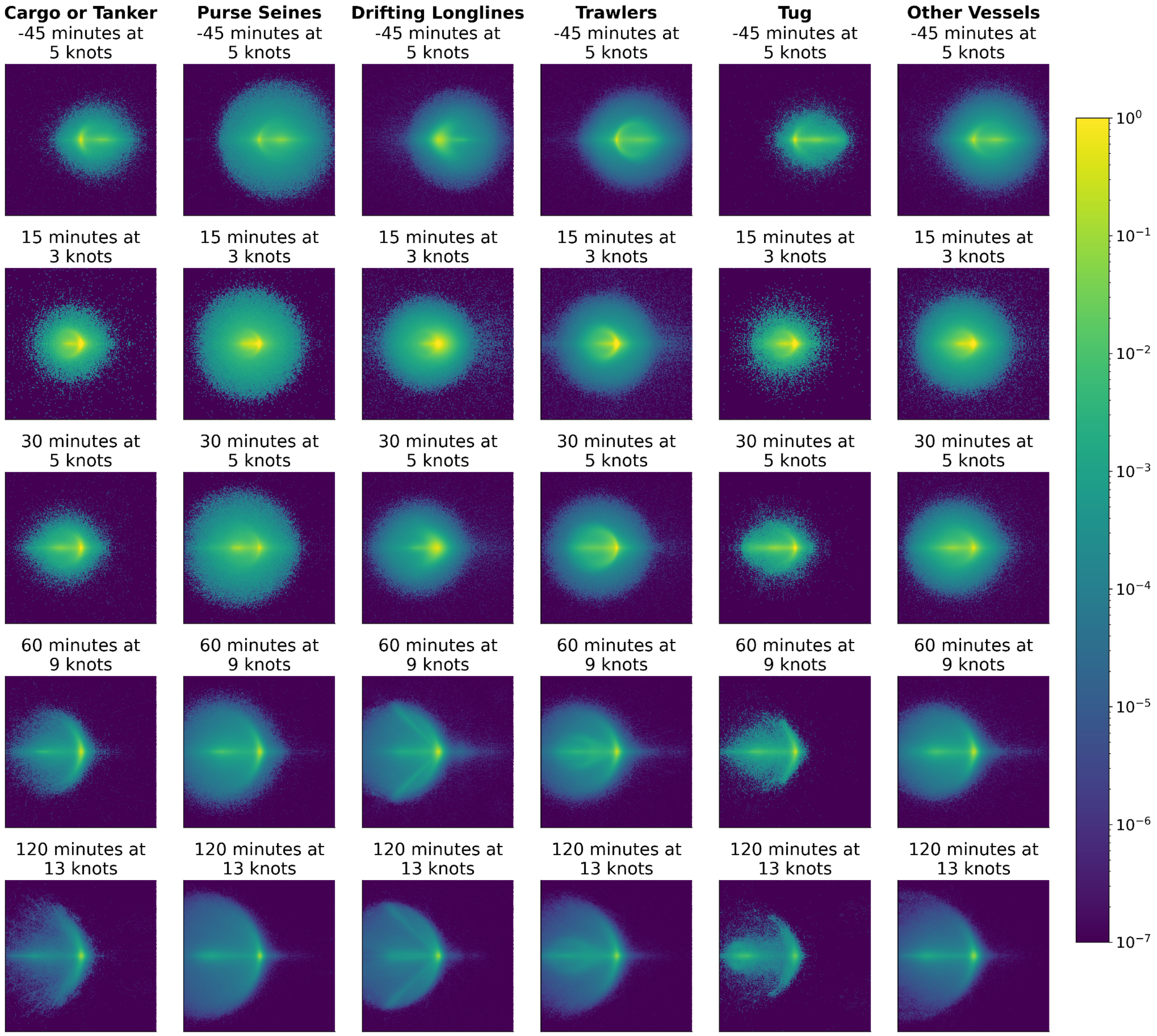

Supplementary Figure S1.

Examples of probability rasters for six different vessel classes for different time intervals and speeds. These probability rasters were used as lookup tables to estimate the likelihood a vessel is at a given location at a given time after the most recent position.

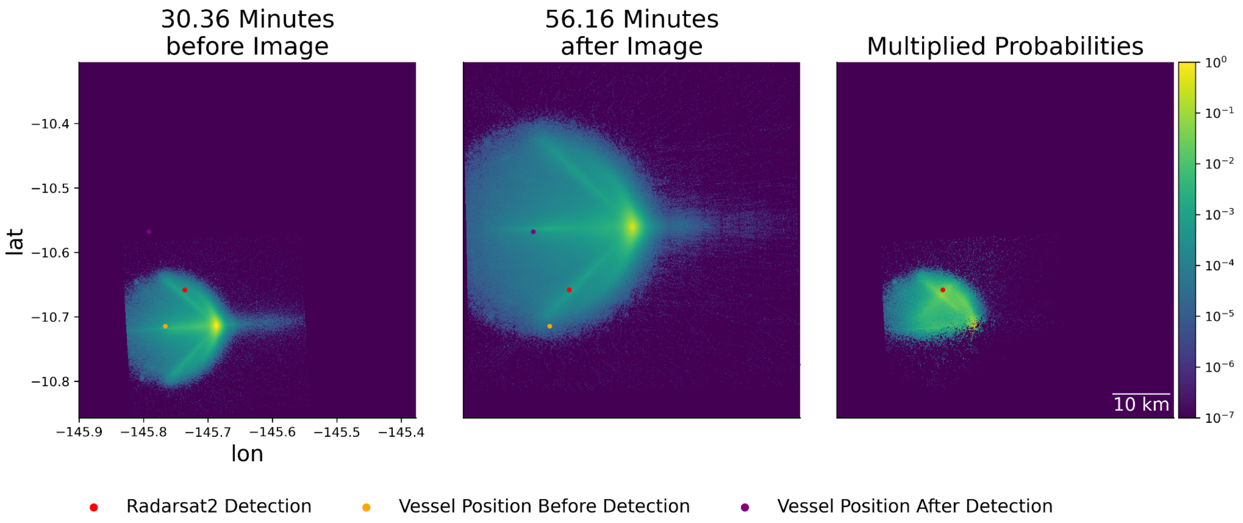

Supplementary Figure S2.
Multiplying probability rasters to match AIS to a SAR detection. For vessels with closest AIS positions more than 10 minutes from the SAR image, the probability rasters were multiplied to estimate a match. Shown are the matches for a vessel with MMSI 577245000. A probability raster is generated for the position before the detection, after the detection, and then the two probabilities are multiplied and renormalized, and this final distribution is compared to the SAR detection (red dot).

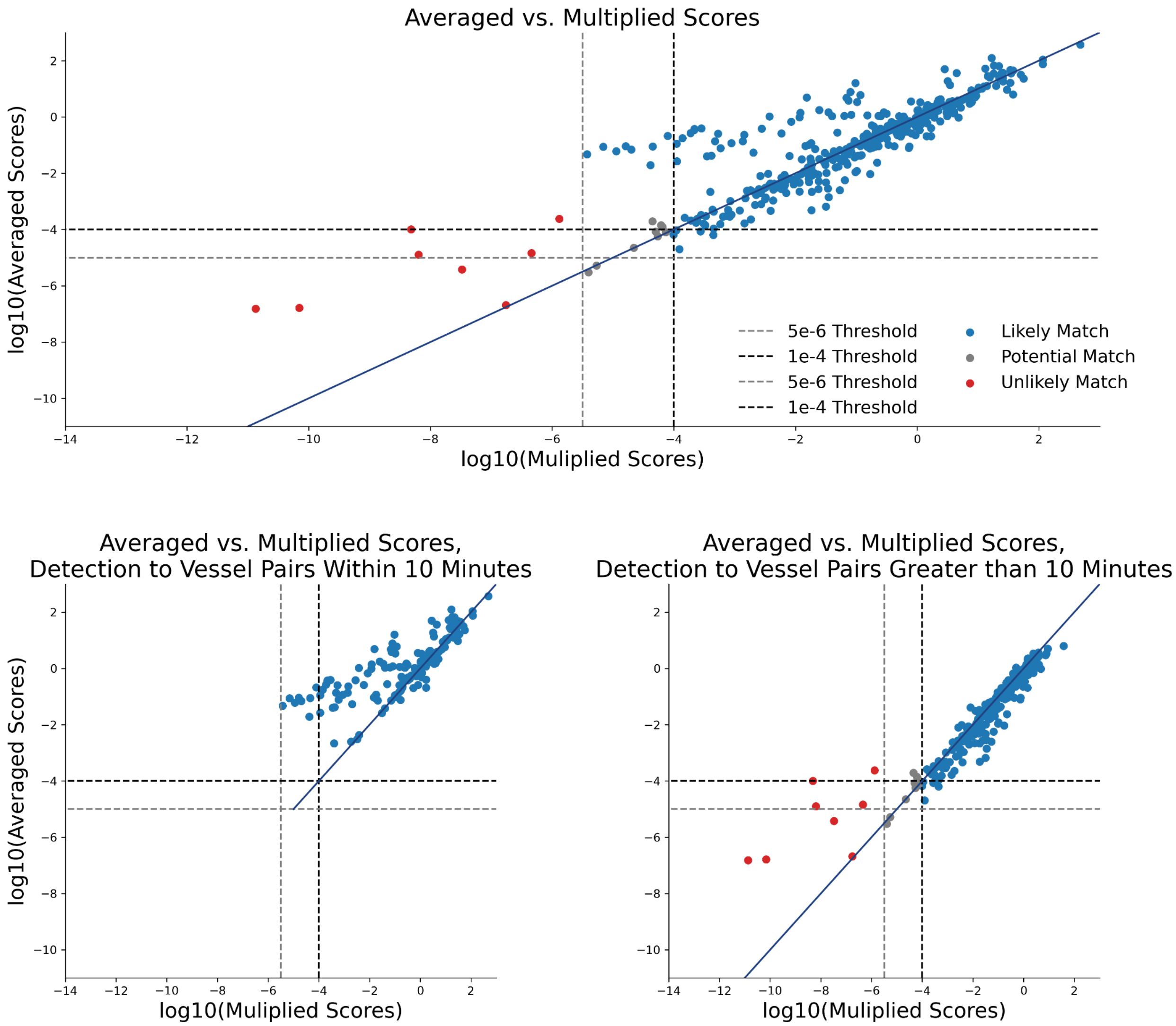

Supplementary Figure S3.

Comparing averaged to multiplied scores and expert judgment. The probability raster for a position before and after a scene can be combined to estimate the probability of a vessel's location by either averaging the values of the two rasters (equation 2) or multiplying (equation 4). All the cases of likely matches (blue) where the multiplied score was much lower than the averaged score were cases in which one of the AIS positions was within 10 minutes of the scene (b and c).

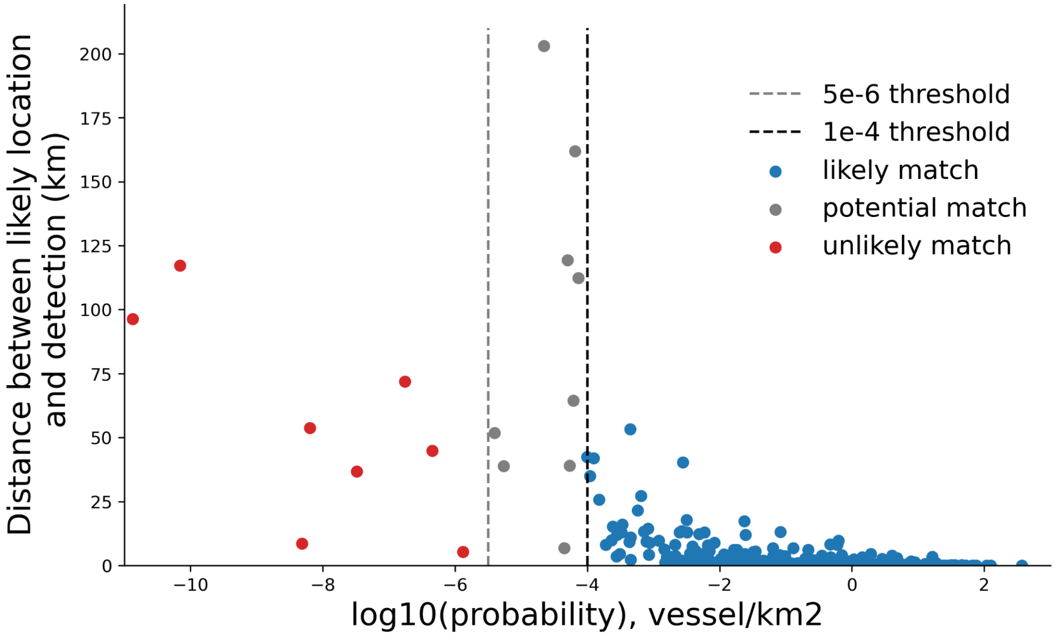

Supplementary Figure S4.
Distance metric compared to synthetic probability. Using the distance between the most likely location of a vessel, based on averaged location (y axis) does not easily identify which AIS-detection pairs should match, as some pairs have a low distance but are highly unlikely according to analyst review (red dots). Our synthetic probability (x axis, log-transformed) well differentiated likely from unlikely matches, with all likely matches above 10^{-4} km^{-2} and potential matches above $5x10^{-6}$ km^{-2}.

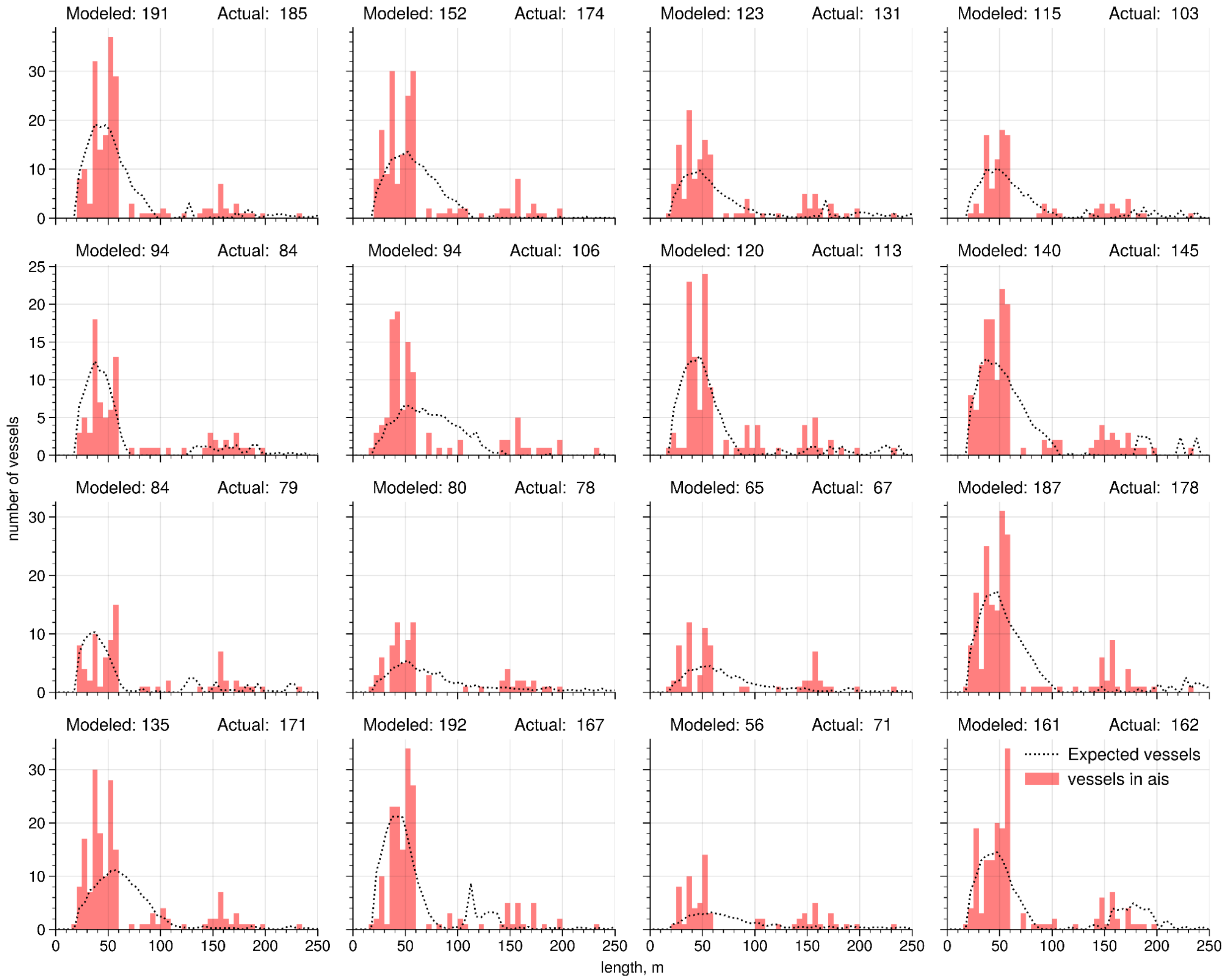

Supplementary Figure S5.

Sixteen example simulations. Random samples of the vessels were used to train the model and estimate the distribution of SAR detections of the remaining vessels. The modeled expected vessels (dotted line; l_E in the methods section of the main text) roughly follow the shape of the actual vessels (red bars; l_O in the methods section of the main text).

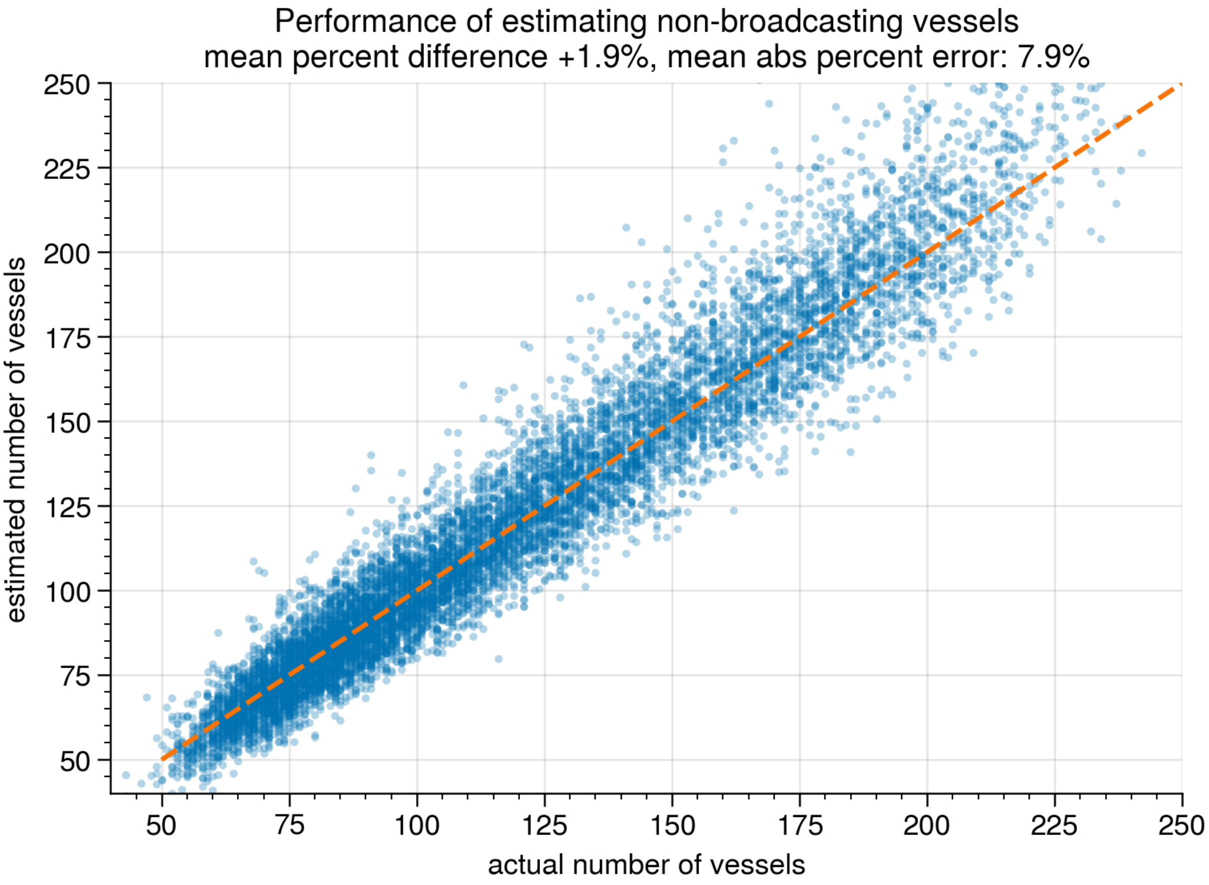

Supplementary Figure S6.
Number of vessels and simulated vessels for 10,000 simulations. Varying fractions of AIS vessels were used to train the model and then estimate the number of remaining vessels based on the SAR detections that match to the remaining vessels and the estimated SAR lengths of these vessels.

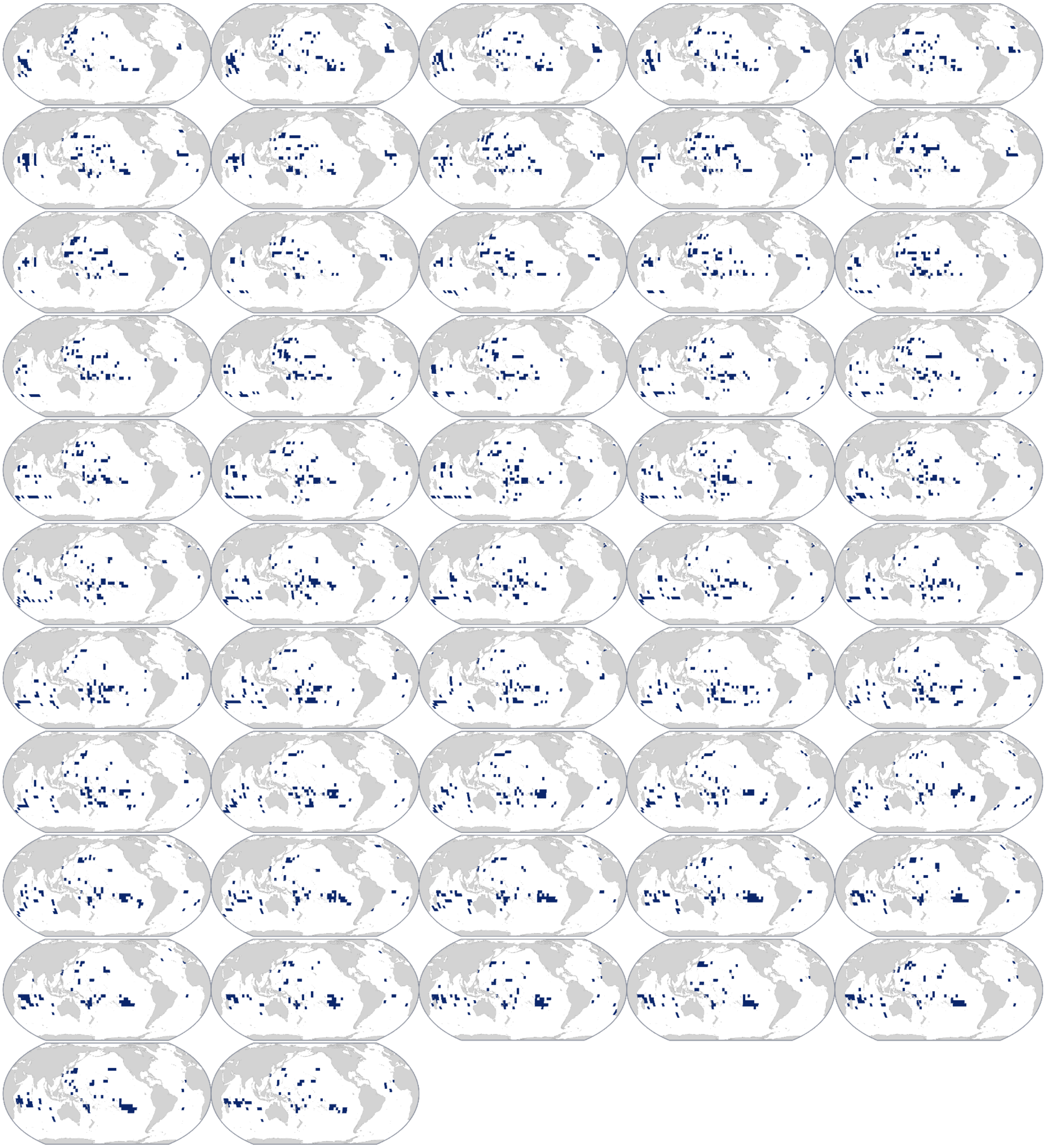

Supplementary Figure S7.
Shown are the areas of the ocean that should be sampled with SAR each week to image about 50% of the pelagic longline activity (based on known AIS longline activity). Each image corresponds to one week, and the colored areas are grid cells to be imaged.

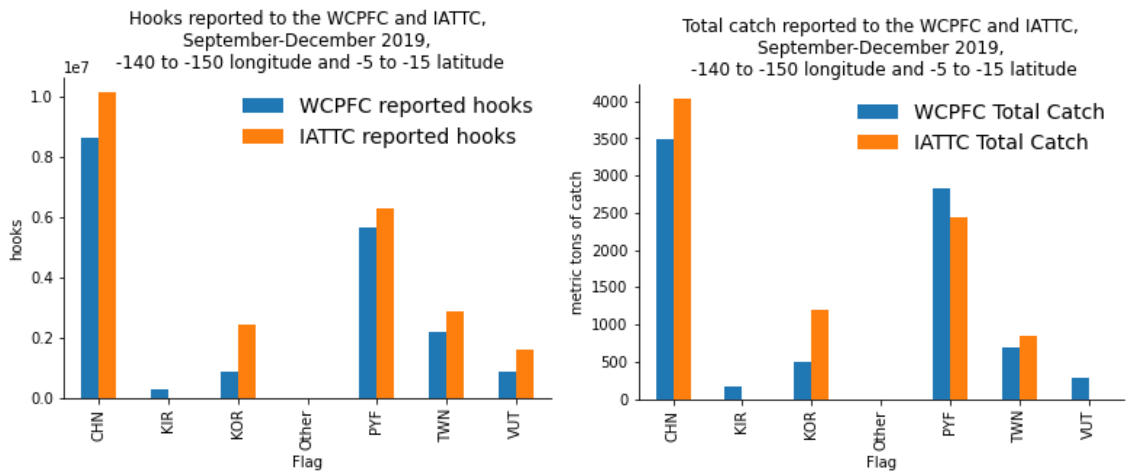

Supplementary Figure S8.

Hooks and catch reported to the Western and Central Pacific Fisheries Commission (WCPFC) and Inter-American Tropical Tuna Commission (IATTC) in the area where these two management organizations have overlapping jurisdiction.

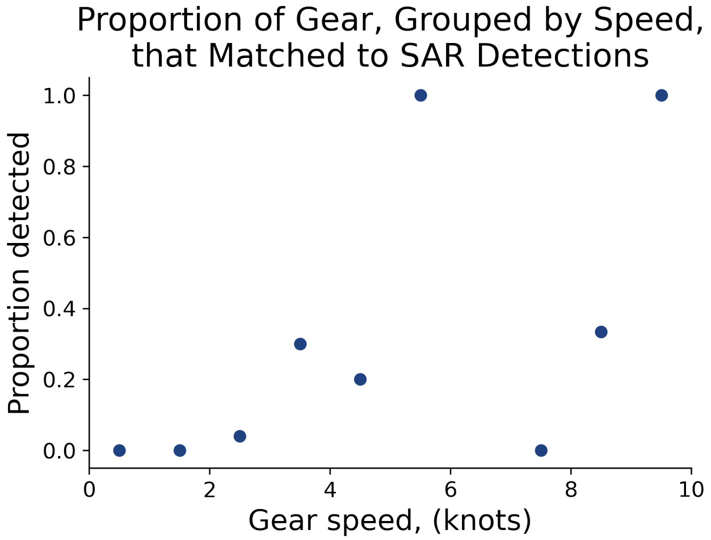

Supplementary Figure S9.

Longline gear with AIS beacons matched to SAR detections only when these AIS beacons were moving faster than two knots, suggesting it was on the deck of a vessel (instead of floating with the current).
